# Mesenchymal stem cells therapy for the treatment of non-union fractures: a systematic review and meta-analysis

**DOI:** 10.1186/s12891-025-08365-w

**Published:** 2025-03-12

**Authors:** Cunbao Cui, Feng Lin, Liang Xia, Xinguang Zhang

**Affiliations:** 1 Department of Joint Surgery, Central Hospital Affiliated to Shandong First Medical University, NO. 105, Jiefang Road, Jinan, 250013 China; 2 Department of Thoracic Surgery, Central Hospital Affiliated to Shandong First Medical University, NO. 105, Jiefang Road, Jinan, 250013 China

**Keywords:** Mesenchymal stem cells, MSCs, Non-union, Bone healing, Meta-analysis

## Abstract

**Background:**

This meta-analysis aimed to pool the existing evidence to determine the clinical efficacy and safety of mesenchymal stem cells (MSC) in patients with non-unions.

**Methods:**

A systematic search in PubMed and Scopus was performed until October 2024 to gather pertinent studies. The inclusion criteria included participants with non-unions, the intervention of MSC administration, a comparator of standard treatment (bone graft), and outcomes focused on healing rate, healing time, or side effects. The Jadad score Newcastle-Ottawa Scale (NOS) was used to assess the risk of bias in randomized and non-randomized studies, respectively. Moreover, GRADE criteria were used to assess the quality of evidence. Using a random effects model, odds ratios (OR) with 95% confidence intervals (CIs) were calculated for healing and complication rates, while standardized mean differences (SMD) with their 95% CIs were used to assess the impact of MSC therapy on bone union time.

**Results:**

Twenty-one studies, with 866 patients, were included. The bone healing rates were 44% at 3 months, 73% at 6 months, 90% at 9 months, and 86% at 12 months, eventually reaching 91% after 12 months of follow-up. MSC therapy, with or without scaffolds, was linked to higher odds of bone healing rate at 3 and 6 months, compared to bone grafts as the standard care (OR = 1.69). The time to union following the treatment was 6.30 months (95%CI: 86-96%), with patients treated with MSC/Scaffold experiencing a shorter time compared to MSC alone (5.85 vs. 6.36 months). MSC therapy significantly decreased bone union time (SMD:-0.54 months, 95% CI: -0.75 to -0.33). The complication rate was 1% (MSC/Scaffold: 0%, MSC alone: 2%), with MSC alone or MSC/Scaffold showing a lower risk than the standard care (OR = 0.41, 95% CI: 0.22–0.78).

**Conclusion:**

MSC is a potential adjunct therapy for patients with non-union fractures.

**Clinical trial number:**

Not applicable.

**Supplementary Information:**

The online version contains supplementary material available at 10.1186/s12891-025-08365-w.

## Introduction

Non-union fractures frequently arise during the healing phase of fractures, affecting approximately 5–10% of all fractures. Managing these cases presents a remarkable challenge given the complexities involved in diagnosing, treating, and achieving favorable patient outcomes [1]. Non-unions are identified as the inability of a fractured bone to achieve bone union within 9 months of the initial injury, with no signs of healing observed for a consecutive period of 3 months [2]. Functional limitations, chronic pain, and an elevated risk of additional injuries associated with non-union fractures have detrimental effects on the physical and mental well-being of patients and result in substantial medical expenses [3–5]. Non-unions can be attributed to various factors, including advanced age, lack of compliance with rehabilitation protocols, malnutrition, diabetes, smoking, immunosuppression, alcoholism, and peripheral neuropathy [6]. In addition, patient-independent factors associated with the fracture site, such as compromised blood supply, the severity of the injury (large gap), surgical osteosynthesis, fracture stability, biomechanics, and infections could contribute to poor healing and increase the risk of non-unions [7, 8].

The economic burden associated with non-union fractures is substantial, leading to significant increases in healthcare costs and resource utilization. It has been reported that the mean total care cost for non-union patients was more than double that of patients without a non-union ($35,317 vs. $102,989) [9]. This financial strain not only impacts healthcare systems but also affects patients’ quality of life due to prolonged recovery times and potential loss of income from missed work [10], highlighting the critical need for effective management strategies for fracture non-unions.

The management of non-union fractures poses a significant challenge for orthopedic surgeons. One major issue is the complexity of the condition itself, as non-unions can arise from various factors, including biological deficiencies and mechanical instability, necessitating tailored treatment approaches for each case [11]. Additionally, the presence of complications such as broken implants from previous surgeries can complicate management strategies and require innovative solutions like combined surgical techniques [12]. The need for meticulous pre-operative planning and a multidisciplinary approach is critical, as improper management can lead to further complications and prolonged recovery times [11]. Furthermore, the high prevalence of non-union fractures, particularly among elderly and osteoporotic patients, adds to the burden on healthcare systems and emphasizes the importance of effective treatment modalities [13].

Currently, the gold standard approach for treating fracture non-unions involves ensuring sufficient stability of the affected area and utilizing autologous bone grafts [14]. Autografts, which involve using the patient’s own bone, have shown union rates ranging from 69 to 89% in various studies [15]. However, complications such as infection and the need for additional surgeries can affect these rates. The overall effectiveness of autografts is attributed to their ability to provide biological support and promote healing, making them a preferred option despite potential drawbacks like donor site morbidity and variability in individual healing responses [16].

Indeed, bone autografts not only serve to fill the gap in non-union cases but also have the potential to enhance the local biology of the fracture site [17]. However, autografts have limitations in their effectiveness. This is particularly evident in cases where there is a reduced availability of endogenous stem cells and their associated bioactive signals within the patient’s bone. These issues are especially prevalent in older individuals or those with certain comorbidities [18, 19].

Consequently, the autograft approach may often fail to promote optimal healing process of nonunions, particularly in cases where there is a reduced availability of endogenous stem cells and their associated bioactive signals within the patient’s own bone, especially in older individuals or those with certain comorbidities [20]. To address this challenge, in recent years, biological therapies, particularly mesenchymal stem cells (MSC), have emerged as a promising approach to improve bone healing and the clinical outcomes of non-union cases [21]. MSC, a type of multipotent stem cells found in various tissues (bone marrow, adipose tissue, placenta, umbilical cord blood), possess remarkable regenerative and immunomodulatory properties [22]. Bone marrow serves as a primary source of MSC [23]. While bone marrow can be utilized directly as Bone Marrow Aspirate Concentrate (BMAC) or processed in vitro to yield Bone Marrow-Derived Mesenchymal Stem Cells (BMSC), BMAC consists of a small proportion of progenitor cells (0.001–0.01%) compared to the more concentrated and purified cell population of expanded BMSC, which belong to the mesenchymal lineage and show promising effects in regenerative medicine and tissue engineering [8]. Their ability to differentiate into osteoblasts, secrete growth factors, and promote angiogenesis makes MSC an attractive candidate for augmenting bone healing processes [24, 25].

Evidence indicates that MSC treatments have a strong safety profile, with most adverse events being mild and manageable [26, 27]. Several studies have investigated the therapeutic potential of MSC in non-unions, demonstrating promising results [28, 29]. However, despite a growing body of literature on the use of MSC in non-unions, the effectiveness and safety of MSC for these patients remain a topic of debate, and the findings of the available studies have been inconclusive. While the study by Hernigou et al. [28] showed that MSC is associated with higher odds of bone healing compared to bone graft, other studies failed to find such an association [30, 31]. Also, the effects on time to bone union is different substantially across studies [32–34]. These differences in the results of studies could be due to the differences in the intervention protocol, sample size, duration of follow-up, and age of participants. Previous meta-analyses included a limited number of studies with small sample sizes and did not comprehensively perform subgroup analyses based on the potential covariates. Furthermore, the available meta-analyses mainly focused on short-term outcomes, neglecting long-term follow-up data that are crucial for assessing the durability of MSC therapy benefits. This meta-analysis aimed to assess the efficacy and safety of MSCs in treating non-union fractures while addressing these gaps by evaluating outcomes across different follow-up durations. Additionally, this study aimed to examine whether the effects are influenced by covariates, such as age, follow-up duration, and intervention protocol.

## Methods

The meta-analysis was conducted following the guidelines outlined in the Preferred Reporting Items for Systematic Reviews and Meta-Analyses (PRISMA) (Supplementary file 1) [35]. Since this review was conducted based on the previously published data, no ethical approval was required to perform the study.

### Search strategy

A comprehensive systematic literature search was conducted in PubMed and Scopus from the inception of each database until October 2024 to obtain pertinent studies with the use of the following search strategy: (“Mesenchymal Stem Cells“[Majr] OR “Mesenchymal Stem Cell*“[Title/Abstract] OR “Mesenchymal Stromal Cell*“[Title/Abstract] OR “Mesenchymal Progenitor Cell*“[Title/Abstract] OR MSCs[Title/Abstract] OR “Bone Marrow Stromal Stem Cells“[Title/Abstract] OR “Bone Marrow Stromal Cell*“[Title/Abstract] OR “Wharton Jelly Cell*“[Title/Abstract] OR “Whartons Jelly Cell*“[Title/Abstract] AND (nonunion*[Title/Abstract] OR non-union*[Title/Abstract] OR “delayed union*“[Title/Abstract] OR “non union*“[Title/Abstract] OR bone graft*[Title/Abstract] OR (union*[Title/Abstract]). No language restriction was considered for the search. Two authors independently reviewed the title/abstract of identified studies to exclude irrelevant studies. In the case of any disagreements, they were resolved through a group discussion. The full texts of the potentially relevant studies were then obtained and assessed based on the eligibility criteria. Subsequently, the references list of the eligible studies underwent a manual screening to identify additional suitable studies.

### Inclusion criteria

To enhance clarity regarding the selection of studies for inclusion in the proportional meta-analysis versus controlled studies, we employed a systematic approach based on predefined eligibility criteria. We established specific inclusion and exclusion criteria that prioritized studies based on their design, methodology, and relevance to the research question, ensuring that both randomized controlled trials (RCTs) and non-randomized studies were assessed for their quality and applicability. Each study underwent a two-phase screening process—initial title/abstract screening followed by full-text evaluation—conducted independently by multiple reviewers to minimize bias and ensure consistency in the selection process. Two independent reviewers (CC and FL) evaluated the eligibility of the studies, and any disagreements were addressed by consulting all the authors. During the group discussion, the two independent reviewers (CC and FL) presented their evaluations of the studies, highlighting the reasons for any disagreements regarding eligibility. The authors collectively reviewed the evidence and rationale provided by each reviewer, engaging in a constructive dialogue to clarify any misunderstandings. This collaborative approach allowed the authors to reach a consensus on the inclusion or exclusion of the studies based on established criteria. Based on the PICOS criteria, the inclusion criteria for this study included: Participants (patients with non-unions), intervention (MSC administration), Comparator (for RCTs, standard treatment (bone graft) was considered as the control, while no restriction for intervention comparison was implied for non-randomized studies), outcomes (healing rate, healing time, or side effects), and study type (clinical studies). The criteria specifically focused on human studies without any date or language limitation and required full-text studies. We included studies with a minimum follow-up period of 3 months to ensure that sufficient time was allowed for evaluating the effects of MSC therapy on bone healing outcomes. Case reports on single patients, reviews, republished studies, protocols, in vitro investigations, animal studies, and studies with irrelevant interventions were excluded. Furthermore, studies involving BMAC, which lacks concentrated MSC, were excluded due to the low MSC concentration and the presence of a blend of progenitor cells.

### Data extraction and quality assessment

The following data were obtained from the included studies: first author, country, year of publication, types of studies, type of MSC, sample size, follow-up duration, time to union, healing rate at 3, 6, 9, 12, and over 12 months following the treatment, type of non-unions, type of fractures, and complications. The process of data extraction was carried out by two independent investigators (CC and FL) and discordances were resolved by a group discussion involving all authors. For randomized clinical trials, the Jadad score, with a range of possible scores of 0 to 5 was used for quality assessment. The Jadad score evaluates four main aspects of the study design, including randomization, blinding, withdrawals, and dropouts [36]. The Newcastle-Ottawa Scale (NOS) was used for assessing the quality of non-randomized studies. The NOS assesses study quality across three main domains, including selection, comparability, and outcome. The NOS uses a star system, with a maximum of nine stars awarded based on criteria met in the three domains, with scores equal to or greater than 7 considered high quality [37]. Moreover, the Grading of Recommendations, Assessment, Development and Evaluation (GRADE) [38] was used to assess the quality of evidence, categorizing evidence into four levels, including high, moderate, low, and very low, based on factors such as risk of bias, inconsistency, indirectness, and imprecision. The quality assessment was conducted by two independent investigators (CC and FL), and any discrepancies were resolved through a group discussion that included all authors.

### Statistical analysis

In studies without a control group, we applied proportional meta-analysis to pool the data. The Effect Sizes (ES) with 95% confidence intervals (CIs) for healing rate (at 3, 6, 9, 12, and over 12 months post-treatment) and complication rate relative to the total number of patients in each study were pooled to estimate the overall effects. In controlled studies, we assessed treatment effects by examining the odds of bone healing and complications as dichotomized outcomes, compared to the standard care, utilizing the odds ratios (OR) and 95% CIs. Moreover, standardized mean differences (SMD) with their 95% CIs were used to examine the effect of MSC on time-to-bone union. Statistical heterogeneity was evaluated by employing the I^2^ statistic, considering it significant when I^2^ exceeded 50% and *p* < 0.10. In case of significant heterogeneity, a random effects model was used for the analyses, otherwise, a fixed effect model was applied. Subgroup analysis by type of intervention (combined therapy with MSC/scaffolds vs. MSC only) and age of participants (≥ 40 years vs. <40 years) was conducted to assess the sources of heterogeneity. Publication bias was measured using Egger’s test. All the analyses were conducted with STATA software (version 13).

## Results

### Characteristics of studies

**Fig. 1:**
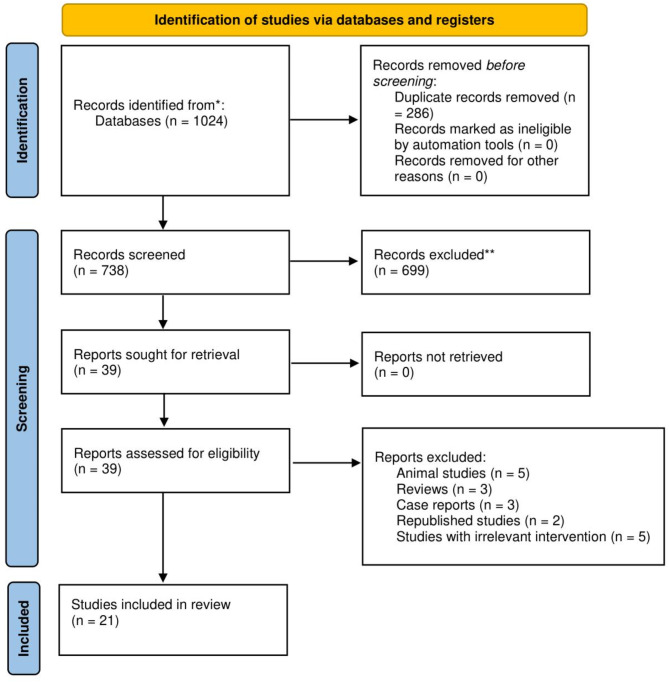
Flow diagram of the study

Out of the initial search of 1024 studies, a total of 21 studies [28–34, 39–52], with a combined sample size of 866 patients, were included (Fig. 1). Of which, there were 8 randomized studies (MSC vs. bone autograft as the standard care) [28, 30–32, 40, 43, 50, 52] and 13 non-randomized studies (received MSC). In all studies, MSCs were injected into the fracture site. For trials with two arms, the intervention group received MSCs combined with autograft and the control group received only autograft, while in non-randomized studies, the intervention consisted of MSCs alone. All studies defined bone healing as bone union, using radiographic follow-ups. The sample size of the included studies ranged from 3 to 307 participants, with a mean age between 5.8 and 77 years. Out of the 21 studies, 6 studies [28, 29, 31, 32, 34, 52] included a scaffold when utilizing MSC. The follow-up duration varied from 3 months to 96 months. The majority of the investigated non-unions involved the tibia/fibula, femur, humerus, and ulna. Healing rate at 3, 6, 9, 12, and over 12 months was presented in 5 [30, 46, 47, 50, 52], 10 [28, 30, 32–34, 42, 46, 47, 49, 50], 4 [31, 34, 39, 50], 7 [30, 32–34, 42, 47, 49], and 10 [29, 30, 34, 40, 41, 43–45, 48, 51] publications, respectively. Moreover, time to bone union was reported in 11 articles [29–31, 33, 34, 40–43, 46, 49]. The characteristics of the eligible publications are presented in Table 1.

**Table 1:** Characteristic of the included studies for the effect of mesenchymal stem cells therapy on non-unions

Study	Year	Country	Type of study	Type of mesenchymal stem cells (MSCs)	Age, mean and SD or range	Sample size	Follow-up duration	Type of Non-union	Type of fracture	Site of fracture	Dose	Main outcomes	Complications	Quality score
Labibzadeh	2016	Iran	Prospective non-randomized study	BMSC	44.7 (26–61)	7 subjects	96 months	7 atrophic	3 closed 4 open	4 Femurs 3 Tibias	For each patient, about 20–50 × 10^6^ cells were injected into the lesions	Time to bone union Healing rate at 6 and 12 months	No complication	4/9
Emadedin	2017	Iran	Prospective non-randomized study	BMSC	32.4 (23–55)	5 subjects	72 months	5 atrophic	5 closed	3 Femurs 2 Tibias	Under fluoroscopic guidance, patients received a one-time implantation of 20–50 × 10^6^ MSCs into the nonunion site	Time to bone union Healing rate at 6 and 12 months	No complication	4/9
Wittig	2016	Venezuela	Prospective non-randomized study	BMSC/Scaffold (collagen microspheres)	50.33 (27–81)	3 subjects	36 months	3 atrophic	NR	1 Femur 2 Tibia	NR	Time to bone union Healing rate at 6, 9, 12, and over 12 months	No complication	4/9
Ismail	2016	Indonesia	Prospective randomized study	BMSC/Scaffold hydroxyapatite granules)	37.6 (18–70)	10 subjects (5 received BMSC/hydroxyapatite + standard care (bone graft); 5 revieved standard care)	37 months	5 atrophic	NR	6 Femurs 2 Tibia 2 Humerus	Treatment group were treated with 15 million autologous BMSCs	Healing rate at 6 and 12 months	No complication	3/5
Hernigou	2021	France	Prospective randomized study	BMSC	40.4 ± 21.2	307 subjects (231 received MSC + standard care (bone graft); 76 recieved standard care)	9 months	all aseptic	All open (98 GIII, 209 GIII)	Tibia	Patients received 9400 ± 1435 MSCs/cc into the nonunion site	Healing rate at 3, 6, and 9 months	Intervention group: 4% (9/231), including superficial infection, minor wound problems, temporary sensory loss, and mild or resolving pain); Standard care: 13% (10/76), including iliac crest infections, large hematomas, and persistent pain)	4/5
Wang	2019	China	Retrospective randomized study	BMSC/ β-tricalcium phosphate scaffolds	36.0 + 14.3	50 subjcts (30 recived BMSC/β-tricalcium phosphate scaffolds + standard care (bone graft); 20 recievied standard care)	9 months	NR	NR	5 Humerus 3 Radius/ulna 23 Femur 19 Tibia/fibula	Approximately 11,444.0 + 6,018 MSCs were injected per patient	Time to bone union Healing rate at 9 months	No complication	4/5
Zhang	2018	China	Prospective randomized study	BMSC	32.69 (19–48)	24 subjects (11 recived BMSC + standard care (bone graft); 13 received standard care)	16 months	NR	NR	Tibia	The MSC harvest was 72 × 10^6^ cells per ml	Time to bone union Healing rate at over 12 months	Intervention group: 27% (3/11) patients reported surgical site infection; Standard care: 15% (2/13) patients reported surgical site infection)	5/5
Dilogo	2019	Indonesia	Prospective non-randomized study	BMSC	29.5 (18–40)	6 subjects	19 months		NR	1 Humerus, 2 Femur 3 Tibia	Patients received 50 million autologous BMSCs contained in 10 mL of plasma solution	Healing rate at over 12 months	No major complication 33% (2/6) subjects developed surgical site infection	4/9
Giannotti	2013	Italy	Prospective non-randomized study	BMSC/scaffold made by autologous plasma gel and CaCl2.	44 (18–73)	8 subjects	50.3 months	Atrophic	12-A1, 12-B3, 12-C1, 21-B1, 22-A1, 22-A2, 22-C3	Upper limb	Patients received 1–4 × 10^6^ cellule/2 ml plasma	Time to bone union Healing rate at over 12 months	No complication	4/9
Centeno	2011	USA	Prospective non-randomized study	BMSC	56.33 (25–82)	6 subjects	9 months	NR	NR	Humerus Tibia	MSCs injected at 30.25 × 10^6^ ± 34.01.	Healing rate at 9 months	No complication	4/9
Dallari	2016	Italy	Retrospective randomized study	BMSC	38.5 ± 2.2	90 subjects (50 recievied BMSC + standard care (bone graft); 40 received standard surgery)	24 months	26 Hypertrophic 64 Atrophic	NR	Tibia or femur	NR	Time to bone union Healing rate at 3, 6, 12, and over 12 months	Intervention group: 6% (3/50) patients developed infection; Standard care: 10% (4/40) patients developed infection	4/5
Flouzat-Lachaniette	2015	France	Prospective non-randomized study	BMSC	49 (22–64)	54 subjects	24 months	NR	Closed	Tibia	The average total number of aspirated MSCs (counted as CFU-Fs) was 74,400 ± 21,300	Time to bone union Healing rate at over 12 months	No complication	8/9
Hernigou	2015	Japan	Prospective randomized study	BMSC/Cancellous chips scaffold	54 (30–80)	172 subjects (86 received BMSC/Cancellous chips scaffold + standard care (bone graft); 86 recived standard care)	6 months	NR	NR	Ankle (pilon tibial, medial malleolar, lateral malleolar, bimalleolar or trimalleolar)	The number of MSCs injected in each non-union was on average 61,000 ± 18,000 CFU-FC	Healing rate at 6 months	Intervention group: 2% (2/86), including temporary sensory loss and mild or resolving pain; Standard care: 11% (10/86), including infection, large haematomas and persistent pain.	5/5
Le Thua	2015	Vietnam	Prospective randomized study	BMSC	35 (17–52)	27 subjects (18 received BMSC + standard care (bone graft); 9 received standard care)	24 months		NR	4 Ulna 2 Humerus 10 Femur 11 Tibia	A total volume of 8 ml BMAC was injected	Time to bone union Healing rate at over 12 months	No complication	5/5
Toro	2019	Italy	Retrospective non-randomized study	BMSC	77 (65–86)	6 subjects	14 months	5 Atrophic 1 Hypertrophic	12.C3 12.C1 12.A1 12.B1	6 Humerus	NR	Time to bone union Healing rate at 3 and 6 months	No complication	9/9
Gómez-Barrena	2020	*Spain*	Prospective non-randomized study	BMSC	39 ± 13	28 subjects	12 months	NR	17 closed 11 open (6 Gustilo I, 5 Gustilo II, III)	11 Femoral 4 humeral 13 Tibial	NR	Healing rate at 3, 6, and 12 months	No complication	8/9
Vugt	2021	*the Netherlands*	Retrospective non-randomized study	BMSC	55 (46–67)	5 subjects	14 months	NR	2 open 3 closed	Tibia	Bone defects were filled with an average 6.2 ml BMAC	Time to bone union Healing rate at 6 and 12 months	40% (2/5) patients developed complications that needed surgical intervention; screw breakage in one patient and a persisting fistula which was debrided and closed during a re-intervention in another patient	9/9
Bhattacharjee	2019	UK	Prospective non-randomized study	BMSC	51 ± 13	35 subjects	32 months	29 Atrophic 6 Hypertrophic	18 open 13 closed 4 unknown	19 Femur 16 Tibia	A mean of 5.5 × 10^6^ BMSCs was inserted into the nonunion site.	Healing rate at over 12 months	One patient (3%) developed sepsis.	6/9
Toosi	2023	Iran	Prospective randomized study	BMSC/collagen/poly glycol acid scaffolds	27.8 ± 5.54	10 subjects (5 received BMSC/collagen/poly glycol acid scaffolds + standard care (bone graft); 5 received standard care)	3 months	NR	NR	Scaphoid	A total volume of 0.5 ml containing 2 million BMACs was injected	Healing rate at 3 months	Intervention group: 40% (2/5) showed complications, including hematoma and transient lateral high paresthesia; Standard care: no complication	4/5
Kurniawan	2023	Indonesia	Prospective non-randomized study	UC-MSC	5.8	6 subjects	29 months	NR	NR	Tibia	NR	Healing rate at over 12 months	One patient (17%) experienced refracture; however, 8 months later, after another implantation and reconstruction were performed, union eventually occurred.	4/9
Dilogo	2021	Indonesia	Prospective non-randomized study	UC-MSC		7 subjects	22 months	NR	NR	1 Tibia 6 Femur	A total of 50 million UC-MSCs was used to fill the bone gap	Healing rate at over 12 months	One subject (14%) developed wound dehiscence and infection	5/9

MSCs: mesenchymal stem cells, BMSC: bone marrow mesenchymal stem cells, UC-MSC: Umbilical cord-derived mesenchymal stem, SD: standard deviation, NR: not reported, CFU-Fs: fibroblast colony-forming units

### Meta-analysis

#### Bone healing at 3 months

**Fig. 2:**
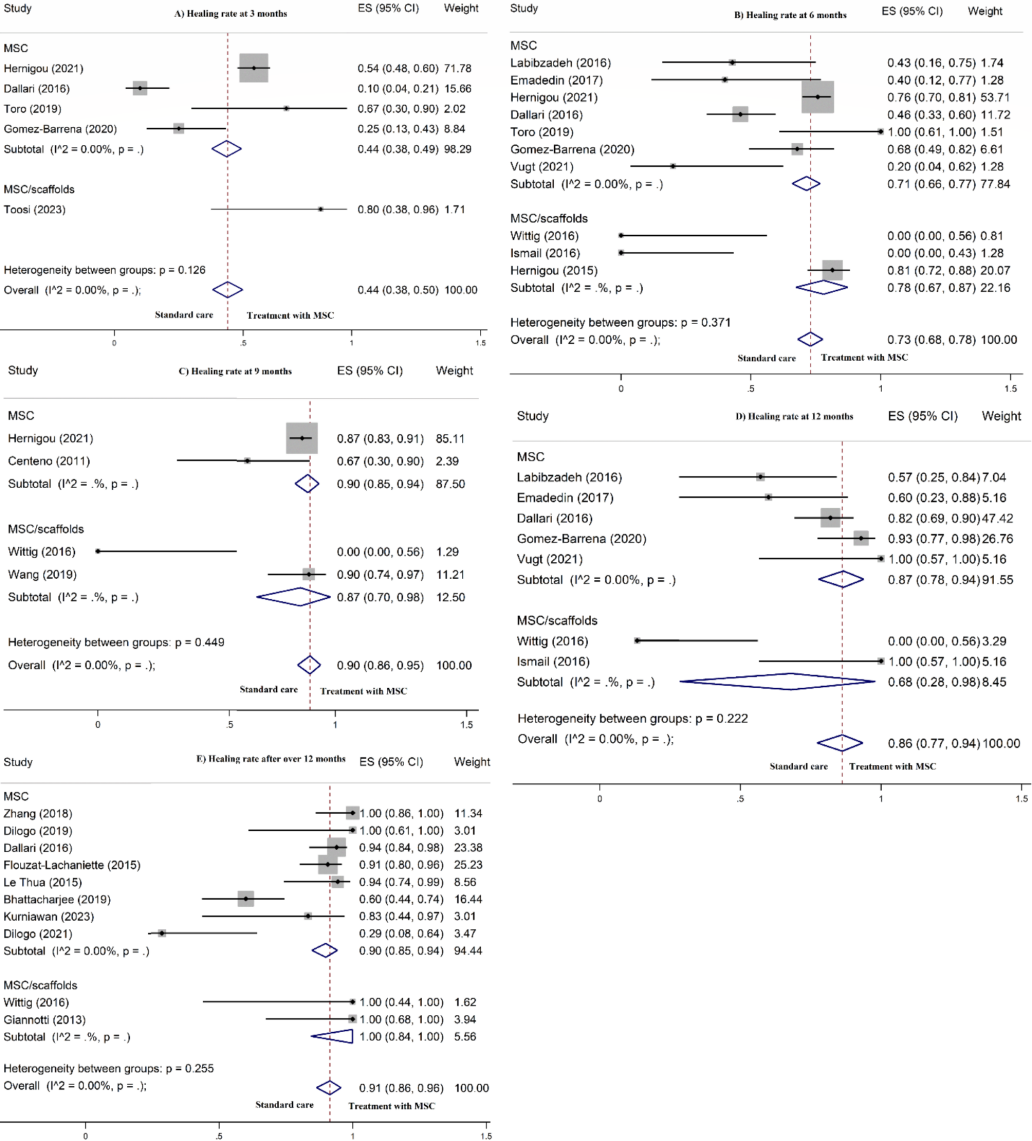
Meta-analysis for the effect of mesenchymal stem cells on bone healing rate at 3 months (**A**), 6 months (**B**), 9 months (**C**), 12 months (**D**), and over 12 months (**E**) post-treatment

**Fig. 3:**
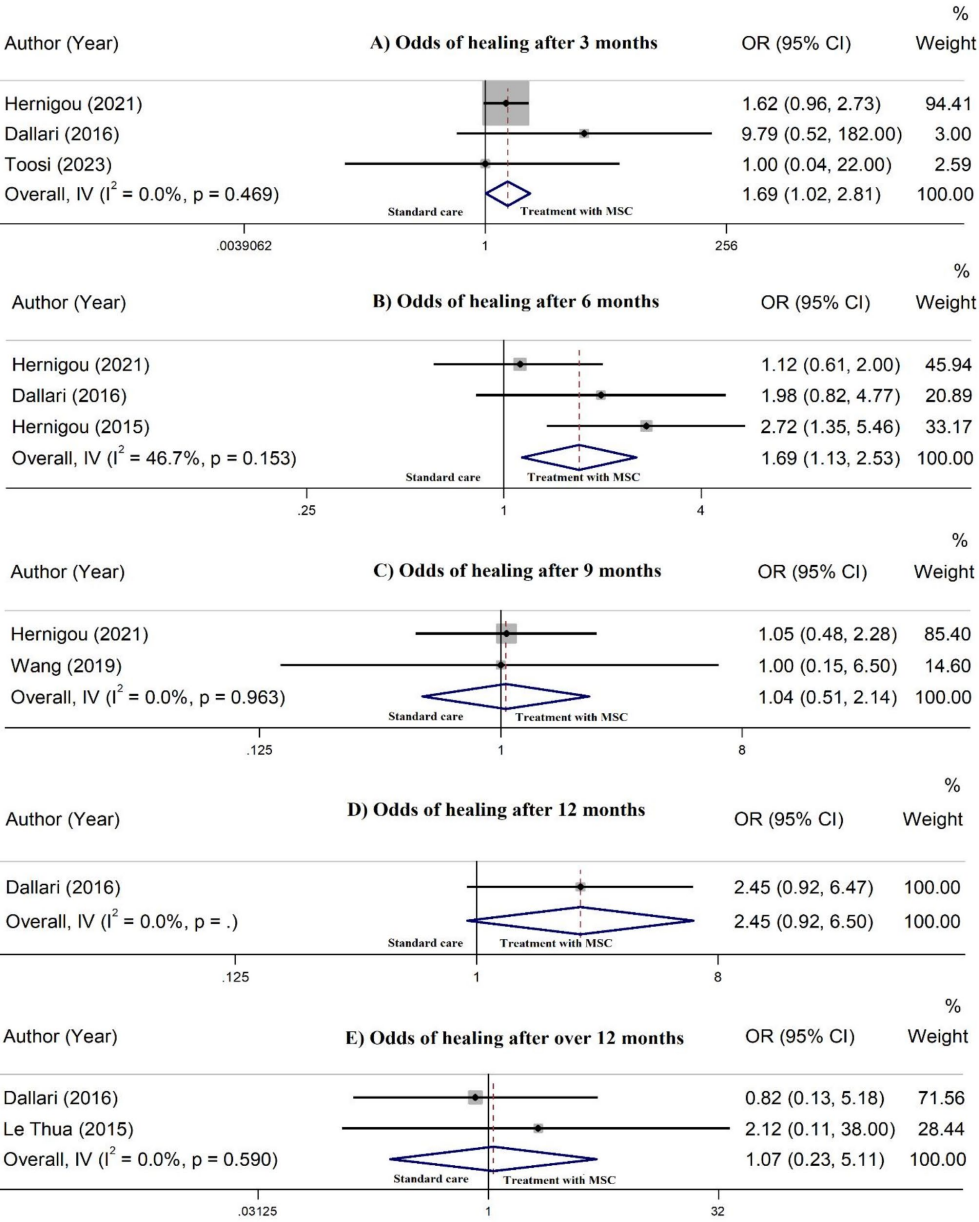
Meta-analysis for the odds of bone healing in patients receiving mesenchymal stem cells, compared to the standard care, at 3 months (**A**), 6 months (**B**), 9 months (**C**), 12 months (**D**), and over 12 months (**E**) post-treatment

Overall, the pooled rate of bone healing was 44.0% (95% CI: 38.0 − 50.0%) after 3 months. Bone healing in patients receiving MSC/Scaffold was 80.0% (95% CI: 38.0 − 96.0%) followed by MSC alone with 44.0% (95% CI: 38.0 − 49.0%). There was no between-group heterogeneity (*P* = 0.12) (Fig. 2). Pooling effect sizes from 3 controlled studies [30, 50, 52] identified that intervention with MSC therapy, with or without scaffold support, is significantly associated with the increased odds of bone healing (OR = 1.69, 95% CI: 1.02–2.81), compared to the standard care (Fig. 3).

#### Bone healing at 6 months

Pooling all effect sizes demonstrated a 73.0% bone healing (95% CI: 68.0 − 78.0%) after 6 months. In patients receiving MSC/Scaffold, bone healing rate was 78.0% (95% CI: 67.0 − 87.0%), while in MSC alone subgroup, the healing rate was 71.0% (95% CI: 66.0 − 77.0%), with no significant between-group heterogeneity (*P* = 0.37) (Fig. 2). Combining effect sizes from 3 controlled studies [28, 30, 50] revealed that MSC therapy, with or without scaffold support, is significantly linked to higher odds of bone healing at 6 months (OR = 1.69, 95% CI: 1.13–2.53), compared to the standard care (Fig. 3).

#### Bone healing at 9 months

In the overall analysis, the rate of bone healing was 90.0% after 9 months (95% CI: 86.0 − 95.0%). In the MSC/Scaffold group, the healing rate was 87.0% (95% CI: 70.0 − 96.0%), while the MSC alone subgroup showed a healing rate of 90.0% (95% CI: 85.0 − 94.0%). No remarkable heterogeneity was found between the groups (*P* = 0.44) (Fig. 2). Meta-analysis of 2 controlled studies [31, 50] revealed no significant distinction in the risk of bone healing (OR = 1.04, 95% CI: 0.51–2.14), compared to the standard care (Fig. 3).

#### Bone healing at 12 months

The analysis indicated that the bone healing rate was 86.0% after 12 months (95% CI: 77.0 − 94.0%). Within the MSC/Scaffold group, the healing rate was 68.0% (95% CI: 28.0 − 98.0%), while the MSC alone subgroup exhibited a healing rate of 87.0% (95% CI: 78.0 − 94.0%). No between-group heterogeneity was found (*P* = 0.22) (Fig. 2). No significant difference in the odds of bone healing was observed (OR = 2.45, 95% CI: 0.92–6.50), compared to the standard care (Fig. 3).

#### Bone healing after over 12 months

Overall, the pooled rate of bone healing was 91.0% (95% CI: 86.0 − 96.0%) after more than 12 months of follow-ups. Bone healing in patients receiving MSC/Scaffold was 100% (95% CI: 84.0 − 100.0%) followed by MSC alone with 90.0% (95% CI: 84.0 − 94.0%). No between-group heterogeneity was detected (*P* = 0.25) (Fig. 2). When the results of controlled studies were pooled [30, 40], no significant difference in odds of bone healing (OR = 1.07, 95% CI: 0.23–5.11) was found compared to the standard care (Fig. 3).

### Time to bone union

**Fig. 4:**
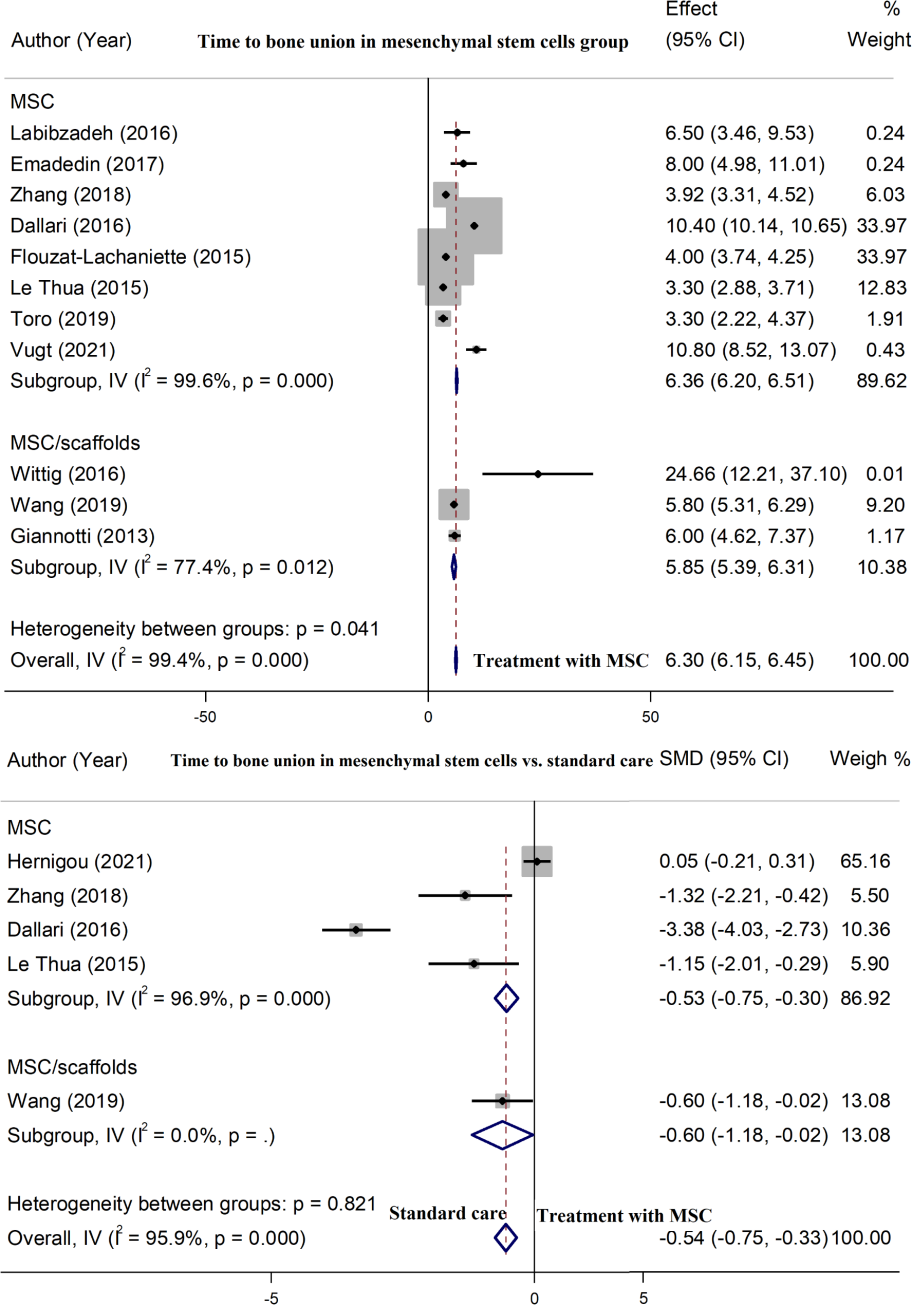
Meta-analysis for time to bone union in patients receiving mesenchymal stem cells (months) (**A**) and in patients receiving mesenchymal stem cells, compared to the standard care (mean difference in months) (**B**)

The mean time to bone union following the stem cell therapy was 6.30 months (95% CI: 6.15–6.45), with a significant heterogeneity (*P* = 0.001). Patients who were treated with MSC/Scaffold had a shorter union time in comparison to MSC alone (5.85 months, 95% CI: 5.39–6.31 vs. 6.36 months, 95% CI: 6.20–6.51) (Fig. 4-A). In contrast, in the group receiving only autograft (without MSCs), the mean time to bone union was 7.78 months (95% CI: 3.17–12.39) (Fig. S1). Pooled analysis from 5 controlled studies [30, 31, 40, 43, 50] showed that stem cell therapy significantly reduced bone union time by 0.54 months (effect size: − 0.54, 95% CI: -0.75 to − 0.33), with remarkable evidence of heterogeneity across the studies (*P* = 0.001). MSC/Scaffold demonstrated superiority over MSC alone in reducing union time (-0.60 months, 95% CI: -1.18 to– 0.02 vs. -0.53 months, 95% CI: -0.75 to– 0.30) (Fig. 4-B).

### Side effects

**Fig. 5:**
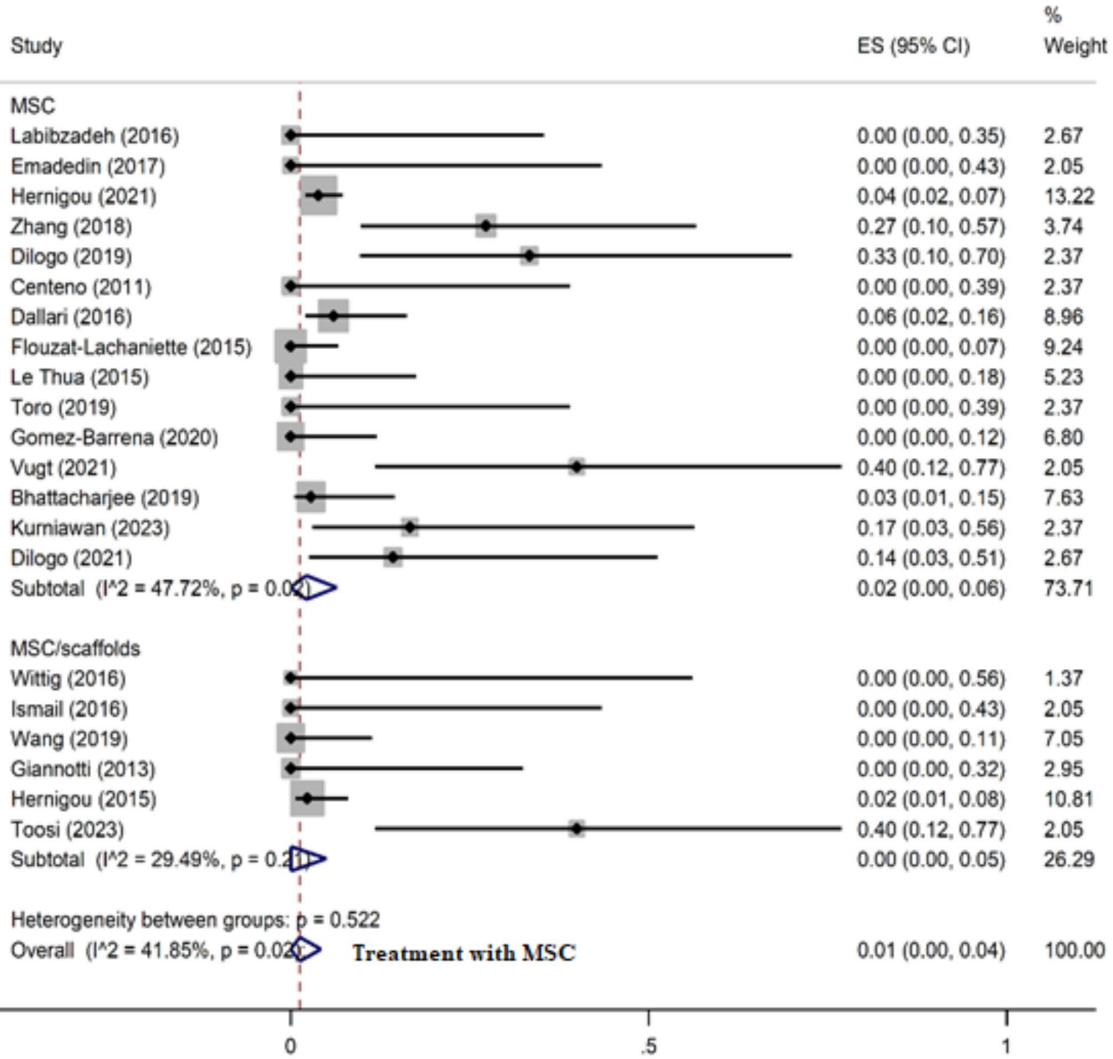
Meta-analysis for complications rate in patients receiving mesenchymal stem cells

**Fig. 6:**
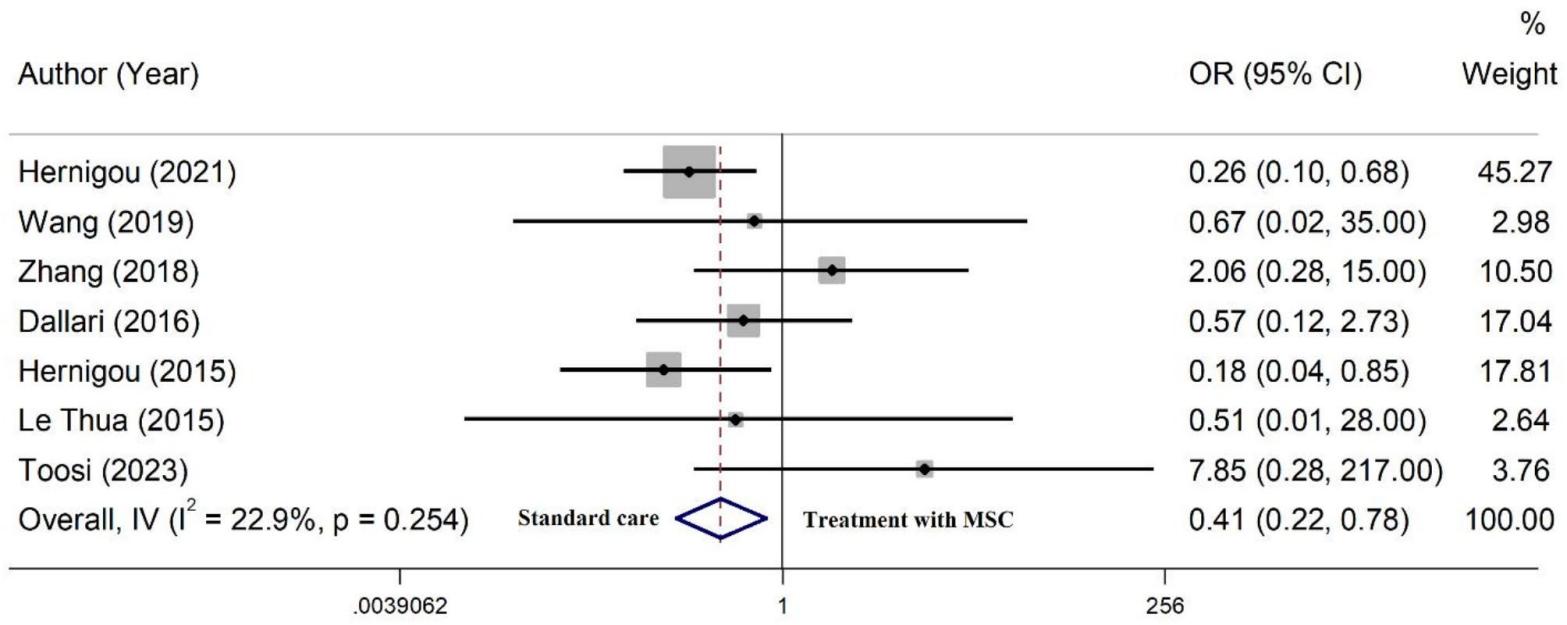
Meta-analysis for the odds of developing complications in patients receiving mesenchymal stem cells, compared to the standard care

Of 21 included studies, 11 studies reported no complications following the treatment, while 10 studies reported some complications [28, 30, 43–45, 48–52]. The most common complication was infection. Other complications included minor wound problems, temporary sensory loss, pain, screw breakage, persisting fistula, hematoma, transient lateral high paresthesia, and refracture (Table 1). The overall complication rate following the treatment was 1.0% (95% CI: 0.0– 4.0%). The rate of complications was lower in patients reviving MSC/Scaffold in comparison to MSC alone (pooled estimate: 0.0%, 95% CI: 0.0 − 5.0% vs. 2.0%, 95% CI: 0.0 − 6.0%) (Fig. 5). In contrast, in the group receiving only autograft (without MSCs), the rate of complications was 8.0% (95% CI: 5– 12%) (Fig. S2). Meta-analysis of 7 controlled studies [28, 30, 31, 40, 43, 50, 52] revealed that treatment with MSC therapy, with or without scaffold support, is significantly associated with a lower risk of developing complications (OR = 0.41, 95% CI: 0.22–0.78), compared to the standard care (Fig. 6).

### Subgroup analysis by age of patients

In older patients (age ≥ 40 years), compared to younger patients (age < 40 years), the bone healing rate was higher at 3 (55.0%, 95% CI: 48.0 − 62.0% vs. 16.0%, 95% CI: 8.0 − 26.0%) and 6 (79.0%, 95% CI: 74.0 − 83.0% vs. 49.0%, 95% CI: 38.0 − 61.0%) months post-treatment, while the rate of healing was comparable at 9 months (91.0%, 95% CI: 86.0 − 95.0% vs. 90.0%, 95% CI: 74.0 − 97.0%) and, in contrast, healing rate was lower for older people at 12 months (63.0%, 95% CI: 34.0 − 83.0% vs. 88.0%, 95% CI: 80.0 − 95.0%) and after over 12 months (86.0%, 95% CI: 77.0 − 93.0% vs. 96.0%, 95% CI: 90.0 − 99.0%) of follow-up. Older patients (age ≥ 40 years), compared to younger patients (age < 40 years), had a shorter time to union (4.13 months, 95% CI: 3.89–4.37 vs. 7.62, 95% CI: 7.43–7.81 months). Moreover, the rate of complications was lower in older patients (age ≥ 40 years), compared to younger patients (age < 40 years) (0%, 95% CI: 0 − 1% vs. 2%, 0% CI: 0%% − 6%).

### Publication bias

**Fig. 7:**
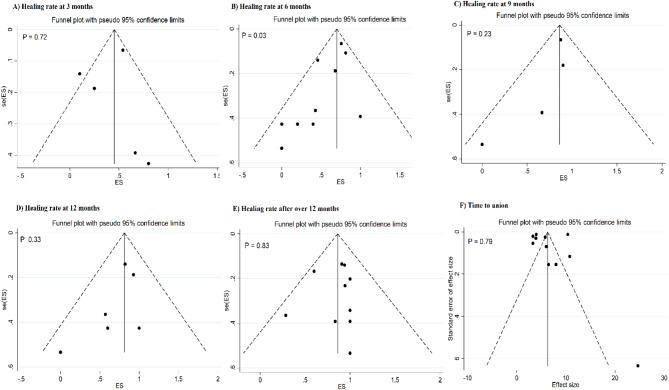
Funnel plot for publication bias for studies on the effect of mesenchymal stem cells on bone healing rate at 3 months (**A**), 6 months (**B**), 9 months (**C**), 12 months (**D**), and over 12 months (**E**) as well as for time to bone union (**F**)

No significant evidence of publication bias was observed for outcomes (Fig. 7).

### Risk of bias and quality of evidence

Regarding the risk of bias, all eight randomized studies included in this meta-analysis were considered as high quality according to the Jadad score, with scores ranging from 3 to 5. In terms of blinding, 2 studies were conducted as open-label studies [28, 50], whereas the blinding status of the other studies remained uncertain. In comparison, the quality of the 13 non-randomized studies varied according to the Newcastle-Ottawa Scale (NOS), with nine studies rated as low quality and four as high quality, yielding scores between 4 and 9 (Table 1). Based on the GRADE criteria, the quality of evidence for the bone healing rate across various follow-up durations was moderate, while the quality for time to bone union was low (Table 2). For all outcomes, no serious risk of bias was observed in the inconsistency or indirectness domains. The quality of evidence for the bone healing rate was downgraded due to significant bias originating from imprecision, which resulted from a low number of studies, small sample sizes, and wide confidence intervals of the effects. Additionally, the quality of evidence for time to bone union was further downgraded due to significant inconsistency detected across the studies.

**Table 2:** Quality of evidence based on the GRADE scale for randomized clinical trials

Quality assessment									Effect		
Outcomes	Design	Number of studies	Risk of bias	Inconsistency	Indirectness	Imprecision	Other considerations	Sample size	Odds ration (95% CI)	SMD (95% CI)	Quality
Bone healing rate at 3 months	Randomized clinical trials	3 studies	No serious	No serious	No serious	Serious^b^	None	407 subjects	1.69 (1.02–2.81)	-	Moderate
Bone healing rate at 6 months	Randomized clinical trials	3 studies	No serious	No serious	No serious	Serious^b^	None	569 subjects	1.69 (1.13–2.53)	-	Moderate
Bone healing rate at 9 months	Randomized clinical trials	2 studies	No serious	No serious	No serious	Serious^b^	None	357 subjects	1.04 (0.51–2.14)	-	Moderate
Bone healing rate at 12 months	Randomized clinical trials	1 study	No serious	NA	No serious	Serious^b^	None	90 subjects	2.45 (0.92–6.50)	-	Moderate
Bone healing rate at > 12 months	Randomized clinical trials	2 studies	No serious	No serious	No serious	Serious^b^	None	117 subjects	1.07 (0.23–5.11)	-	Moderate
Time to bone union	Randomized clinical trials	studies	No serious	Serious^a^	No serious	Serious^b^	None	498 subjects		SMD = − 0.54, (95% CI: -0.75 to -0.33)	Low

NA: not applicable, SMD: standardized mean difference

^a^ Significant heterogeneity

^b^ Low number of studies, low sample size, and wide confidence interval

## Discussion

The treatment of long bone non-unions is still a major clinical and socio-economical problem. Autologous bone grafts (ABGs) have long been regarded as the standard care for treating non-unions, particularly due to their effectiveness in promoting bone healing and regeneration [14]. This approach involves harvesting bone tissue from the patient, typically from sites such as the iliac crest, and transplanting it to the non-union site. ABGs are favored due to their unique properties, which include osteogenic, osteoinductive, and osteoconductive capabilities [53]. This means ABGs not only provide a scaffold for new bone growth but also contain living cells and growth factors that actively promote healing. Despite their effectiveness, ABGs have some limitations, including the availability of donor tissue and potential complications at the donor site [54].

This meta-analysis was conducted in response to the inconclusive findings from recent studies that assessed the clinical efficacy of MSC administration in patients with non-union fractures. The healing rates observed after MSC therapy, 44% at 3 months, 73% at 6 months, 90% at 9 months, and 91% after 12 months, indicate a progressive improvement in bone healing, which is clinically significant as it suggests that patients may experience quicker recovery and return to normal activities. The odds ratios (OR = 1.69) at both 3 and 6 months further emphasize the enhanced odds of successful healing compared to standard care, translating to improved patient outcomes and reduced risk of complications. However, the lack of significant differences in healing rates beyond 6 months, compared to standard care, suggests that while MSC therapy is beneficial in the short term, long-term outcomes may require additional interventions or monitoring to ensure sustained recovery. The short-term benefits of MSCs in bone healing rates, compared to allografts, can be attributed to their unique biological properties and the dynamic nature of the healing process. Initially, MSCs enhance osteogenesis and promote rapid tissue regeneration through their ability to differentiate into osteoblasts and chondrocytes, which are essential for forming the bone matrix and cartilage during early healing stages [55, 56]. Additionally, MSCs secrete various cytokines that modulate inflammation and promote angiogenesis, creating a favorable microenvironment for bone repair [57]. However, as healing progresses into the long term, the initial advantages provided by MSCs may diminish due to factors such as senescence of transplanted cells, altered local microenvironments, and potential competition with endogenous stem cells or other repair mechanisms that may not rely on MSCs [58]. Thus, while MSCs are highly effective in the early phases of bone healing, their long-term efficacy may be limited by these biological dynamics and the complexity of the bone remodeling process.

Patients receiving MSC/Scaffold generally exhibited higher healing rates, compared to those receiving MSC alone. Notably, the MSC/Scaffold group showed improved bone healing rates at 3, 6, and after more than 12 months, while the MSC alone subgroup demonstrated comparable or slightly higher rates at 9 and 12 months. These results suggested that MSC/Scaffold may have a more significant impact on bone healing, with both treatment groups showing substantial healing rates over time. The results also indicated that intervention with MSC/Scaffold, or MSC alone, significantly reduced overall bone union time, with MSC/Scaffold proving more effectiveness than MSC alone in reducing union time, showcasing a potential advantage of this combined therapy. Furthermore, we identified that MSC therapy, especially with scaffold support, may potentially lower the risk of developing complications in these patients.

Studies indicate that age significantly impacts bone healing rates and times, with older individuals experiencing delayed healing due to decreased function of MSCs and alterations in inflammatory responses [59]. Subgroup analysis based on the age of patients revealed interesting trends. The findings suggested that while older patients may have higher bone healing rates in the short term (6 months), they may exhibit a lower healing rate in the long term (after 12 months). Older patients also showed shorter time to unionand fewer complications. These findings suggest that age may play a significant role in bone healing outcomes and complication rates following MSC treatment for non-union bones. These observations propose that MSC may be more effective in promoting initial healing in older patients, but the long-term healing outcomes may be better in younger patients. Physiological differences due to age-related changes in bone metabolism, regenerative capacity, and healing processes may influence the initial response to treatment, leading to higher short-term healing rates in older patients [60]. Older patients may have fewer complications due to better management of comorbidities or a more controlled healing environment. The slower long-term healing in older patients could be attributed to age-related factors affecting the later stages of bone repair and remodeling [61, 62]. MSC obtained from older donors have demonstrated a decline in proliferative abilities, differentiation potential, and migratory capacity, which could affect the bone healing process [63]. Aging is associated with increased levels of inflammatory cytokines, which can disrupt the balance between bone formation and resorption [64]. This chronic low-grade inflammation may impair the regenerative capacity of MSCs and other bone cells, leading to a lower healing rate [65]. The interplay between MSCs and macrophages also shifts with age, affecting how well these cells can coordinate the healing process [66]. These findings highlight the need for tailored treatment strategies that consider age-related factors when utilizing MSC therapy for non-union fractures. Understanding these dynamics can guide clinicians in optimizing patient management and setting realistic expectations for recovery timelines. Given that older patients may have fewer complications but lower long-term healing rates, it may be beneficial to develop specific protocols that address these differences. For example, enhancing the regenerative environment for older patients through adjunct therapies or modifying MSC treatment approaches could improve long-term outcomes. These results highlight the complex interplay of age-related factors in bone healing in response to MSCs, emphasizing the necessity for further research to elucidate the underlying mechanisms involved.

The clinical trial study by Gómez-Barrena et al. evaluated the efficacy of autologous bone marrow-derived MSCs combined with bioceramics for healing long bone delayed unions and non-unions in 28 patients. Radiological consolidation rates improved significantly over time, reaching 92.8% at 12 months, with histopathological confirmation of bone formation. Non-smokers exhibited better consolidation outcomes compared to smokers, highlighting the influence of lifestyle factors on bone healing [47]. In another study, the long-term efficacy and safety of autologous human MSCs embedded in fibrin clots for treating upper limb non-unions were examined. Eight patients received ex vivo expanded MSCs with no evidence of tissue overgrowth or tumor formation, achieving successful clinical outcomes and restored limb function over an average follow-up of 76 months. The findings supported the use of this entirely autologous approach as a safe and effective treatment for bone non-unions [67]. The effect of MSC on non-unions has been investigated as a secondary outcome in two small meta-analyses [8, 68], both of which, in contrast to the present study, showed no significant association between MSC therapy and odds of bone healing, compared to the standard care. Previous meta-analyses [8, 68] included a small number of studies (3 and 8 studies), included case reports on single patients, combined the results of animal studies with human studies [8, 69], did not assess the complications comprehensively, and did not perform subgroup analysis based on the age of participants, which reduce the reliability of their findings and prone them to the risk of bias. The lack of significant association between adjunct therapy with MSC and risk bone healing in previous studies may be due to their small sample size and lack of statistical power to detect the associations.

Supporting our findings, there is evidence that using MSC in combination with a scaffold may have an advantage over MSCs alone to improve clinical outcomes of non-unions [8]. Appling bone scaffolds is crucial for enhancing the engraftment of implanted progenitor cells. These scaffolds provide a supportive framework that mimics the natural structure of bones, facilitating the integration of the implanted cells with the surrounding tissue [70]. By offering mechanical support, guiding cell growth, and promoting vascularization, bone scaffolds play a vital role in promoting cell attachment, proliferation, and differentiation [71]. This, in turn, improves the overall success of progenitor cell-based therapies in bone regeneration and repair [72]. The application of tissue-engineered scaffolds together with stem cell technologies, particularly MSCs, is believed to hold great potential for bone tissue engineering and regenerative medicine. These scaffolds, in combination with stem cells, are aimed at inducing bone regeneration through the synergistic integration of biomaterial scaffolds, bone progenitor cells, and bone-forming factors [73]. The clinical utilities of the present study include providing a promising treatment approach, reducing recovery time, improving union rates, offering potential non-surgical options, and inspiring further advancements in the field of MSC-based therapies for non-unions. The combination of MSCs and scaffolds improves the biological and mechanical environment necessary for effective bone healing. This synergy not only promotes osteogenesis but also improves the mechanical stability of the fracture, reducing strain and allowing for the gradual transition from soft to hard callus formation [74]. Compared to standard treatments like autografts, which often involve invasive surgical procedures with higher complication rates, the MSC-scaffold approach minimizes surgical intervention and associated risks while potentially improving patient outcomes through enhanced healing capabilities. This method emphasizes the importance of combining biological factors with mechanical stability to address non-union effectively.

The findings of this meta-analysis have some implications for health-care providers and policy makers. The demonstrated efficacy of MSC therapy in improving bone healing rates and reducing time to union suggests that healthcare authorities could consider integrating MSC treatments into clinical guidelines for managing non-union fractures. This could enhance treatment options available to patients, particularly those who do not respond well to standard treatments like bone grafting. Given the positive outcomes associated with MSC therapy, policymakers may need to evaluate funding and reimbursement frameworks for regenerative medicine. This includes establishing reimbursement protocols for MSC therapies, which could encourage wider adoption among clinicians and increase patient access to innovative treatments. The variability in MSC application (with or without scaffolds) and the observed differences in healing times highlight the need for standardized treatment protocols. Policymakers should advocate for multi-center trials that establish best practices for MSC administration, ensuring consistency in treatment approaches across different healthcare settings. To facilitate the adoption of MSC therapies, educational initiatives should be implemented to inform healthcare providers about the latest advancements in stem cell research and its applications in treating non-unions. This could enhance clinician confidence in utilizing these therapies effectively. Moreover, incorporating MSC therapy into treatment plans can shift care models towards more patient-centric approaches, where individualized treatment options are prioritized based on specific patient needs and responses to prior interventions.

The favorable effects of MSCs on non-union healing can be attributed to several biological mechanisms. MSCs possess the ability to differentiate into various cell types involved in the bone healing process, such as osteoblasts, chondrocytes, and endothelial cells; by differentiating into these cell types, MSCs contribute to the formation of new bone tissue, cartilage, and blood vessels, thereby promoting the healing process [24, 25]. MSCs release a variety of bioactive molecules, including growth factors, cytokines, and chemokines [75]. These molecules have immunomodulatory properties and can regulate the inflammatory response at the injury site [22]. By modulating inflammation, MSCs create an environment that is conducive to tissue regeneration and healing [76]. Additionally, MSCs possess paracrine effects, where they secrete extracellular vesicles containing various signaling molecules. These vesicles can stimulate nearby cells, promoting their proliferation, migration, and differentiation, which are all crucial steps in the healing process [77, 78]. MSCs have been also shown to enhance angiogenesis and the formation of new blood vessels. This is particularly important for non-unions, as the presence of a sufficient blood supply is crucial for the delivery of oxygen, nutrients, and cells necessary for efficient healing [79].

Regarding the safety of MSCs in nonunion cases, the majority of the included studies reported no complications. The overall complication rate was low at 1.0%, and treatment with MSCs combined with scaffolds showed a lower complication (0.0%) rate compared to MSCs alone (2.0%), with infection being the most common, highlighting the potential of this approach to improve patient outcomes while minimizing risks associated with traditional grafting techniques. In contrast, in the group receiving only autograft (without MSCs), the rate of complications was remarkably higher (8.0% vs. 1.0%). The lower complication rate observed with the combination of MSCs and scaffolds, compared to MSCs alone, can be explained by several factors related to scaffold properties and their interaction with MSCs. Scaffolds provide a three-dimensional structure that supports cell attachment, proliferation, and differentiation, which enhances the retention of MSCs at the injury site and promotes a more organized tissue regeneration process [80]. This structural support can help mitigate complications such as infection, which was the most common adverse event noted in MSC treatments alone [56]. In contrast, MSCs without scaffolding may disperse too quickly from the site or fail to integrate effectively into the healing tissue, leading to higher complication rates [81]. Additionally, meta-analysis indicated that MSC therapy is significantly associated with a reduced risk of complications compared to standard care. In line with our findings, evidence indicates that MSC treatments have a strong safety profile, with most adverse events being mild and manageable [26, 27].

This study represents the most comprehensive meta-analysis of prior studies on the clinical efficacy and safety of MSCs in non-union fractures. The strengths of this study include its comprehensive analyses based on various follow-ups, absence of publication bias, and subgroup analyses based on the intervention protocol and age of participants, allowing for a more reliable conclusion.

However, it is important to consider the limitations of this meta-analysis. The quality of evidence was low for time to bone union, primarily due to imprecision, because confidence intervals included null effect or appreciable harm or benefit, which resulted from the small sample size and significant inconsistency across studies. Thus, these results should be approached with caution and verified in future well-designed RCTs. Furthermore, we did not register the protocol for this study, which should be considered as a limitation. A significant heterogeneity was observed for some analyses. The stratified analysis revealed that the differences in study intervention (MSC alone vs. MSC /scaffolds), follow-up durations, and age of participants were sources of the observed heterogeneity. In addition, the majority of included articles were non-randomized studies, which can potentially introduce bias. It is important to acknowledge the risk of bias inherent in the included studies, particularly among the non-randomized studies, which may introduce systematic biases in the results. Additionally, the heterogeneity in study designs, including variations in interventions, follow-up durations, and participant characteristics, complicates the interpretation of pooled data may affect the generalizability of our findings. The included studies also did not provide detailed information on smoking, fracture type, and comorbidities, such as diabetes or cardiovascular conditions, which are known to influence healing outcomes and complications. As a result, we were unable to perform a subgroup analysis based on these confounding factors to assess their effects on heterogeneity. This lack of reporting highlights a significant gap in the literature, highlighting the need for future studies to standardize the reporting of environmental risk factors, comorbidities, and fracture classifications. The dosages of MSCs utilized in each study and the protocols for isolating and preparing MSCs varied among the studies. These differences could contribute to heterogeneity across the studies. However, this is a common limitation in meta-analyses involving MSCs, given that there is no standardized protocol for MSC preparation and administration across different studies. Accordingly, standardizing the protocols for isolating and administering MSCs could help reduce heterogeneity among the studies.

Identifying potential sources of heterogeneity, such as differences in MSC interventions, follow-up durations, and participant age, strengthens the analysis by allowing for a more nuanced understanding of treatment effects across diverse patient populations. This stratification can help identify specific factors that influence outcomes, thereby enhancing the robustness of the conclusions drawn from the meta-analysis. Furthermore, recognizing the predominance of non-randomized studies highlights the need for caution in interpreting results and underscores the importance of future randomized controlled trials to mitigate bias. Nevertheless, the presence of significant heterogeneity suggests that the results may not be universally applicable to all patients with non-union fractures. For instance, older patients with fewer comorbidities might respond differently to MSC therapy compared to younger patients or those with underlying health issues. Clinicians must consider MSC types, dosing, age, and follow-up duration when interpreting the findings and making treatment decisions.

In conclusion, this meta-analysis indicated the effectiveness of adjunct therapy with MSCs, either alone or as MSCs/scaffold, in accelerating fracture union time and enhancing fracture union rate in the healing process of bones, especially in older patients. Overall, the use of MSCs was safe; however, there was weak evidence suggesting a risk of post-treatment infections following MSCs therapy. Therefore, this study suggests that combining MSCs with surgical techniques in patients with non-unions can lead to positive clinical outcomes. Future research, particularly with a randomized clinical trial design, is needed to explore the optimal MSC types, dosing, and long-term follow-up protocols in these patients.

## Electronic supplementary material

Below is the link to the electronic supplementary material.

Supplementary file 1

Supplementary figures

## Data Availability

No datasets were generated or analysed during the current study.
