# Supplementary material for: Mesenchymal stem cells therapy for the treatment of non-union fractures: a systematic review and meta-analysis
Source: BMC Musculoskelet Disord. 2025 Mar 12;26:245. doi: 10.1186/s12891-025-08365-w (PMC11900535; doi:10.1186/s12891-025-08365-w)
Supplement: Supplementary file 2 — Supplementary figures [file 12891_2025_8365_MOESM2_ESM.docx]

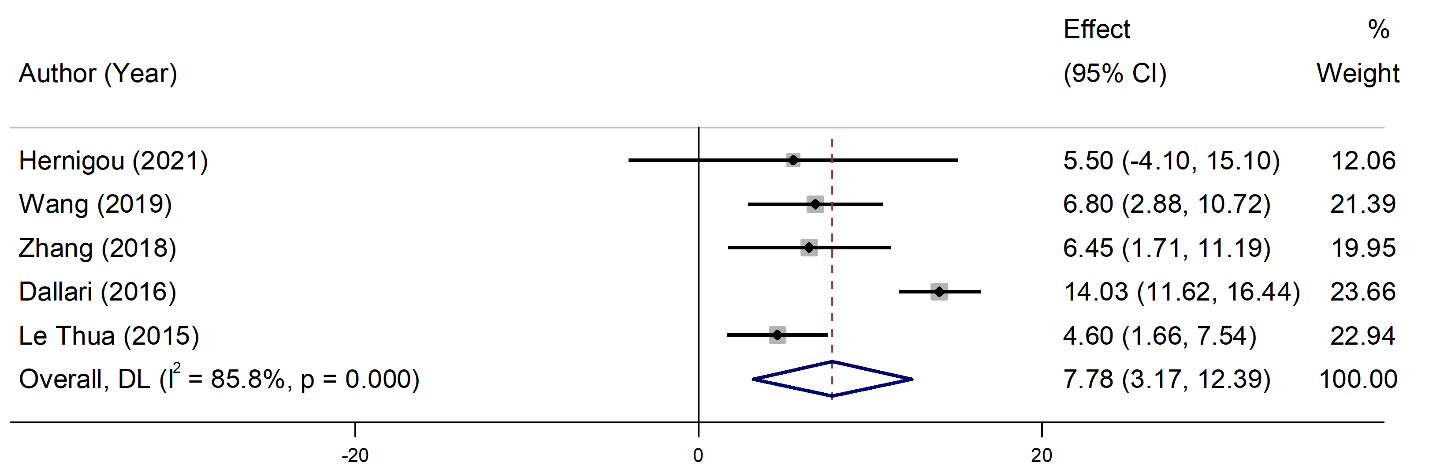


Fig. S1. The mean time to bone union in the group receiving only autograft (without MSCs)


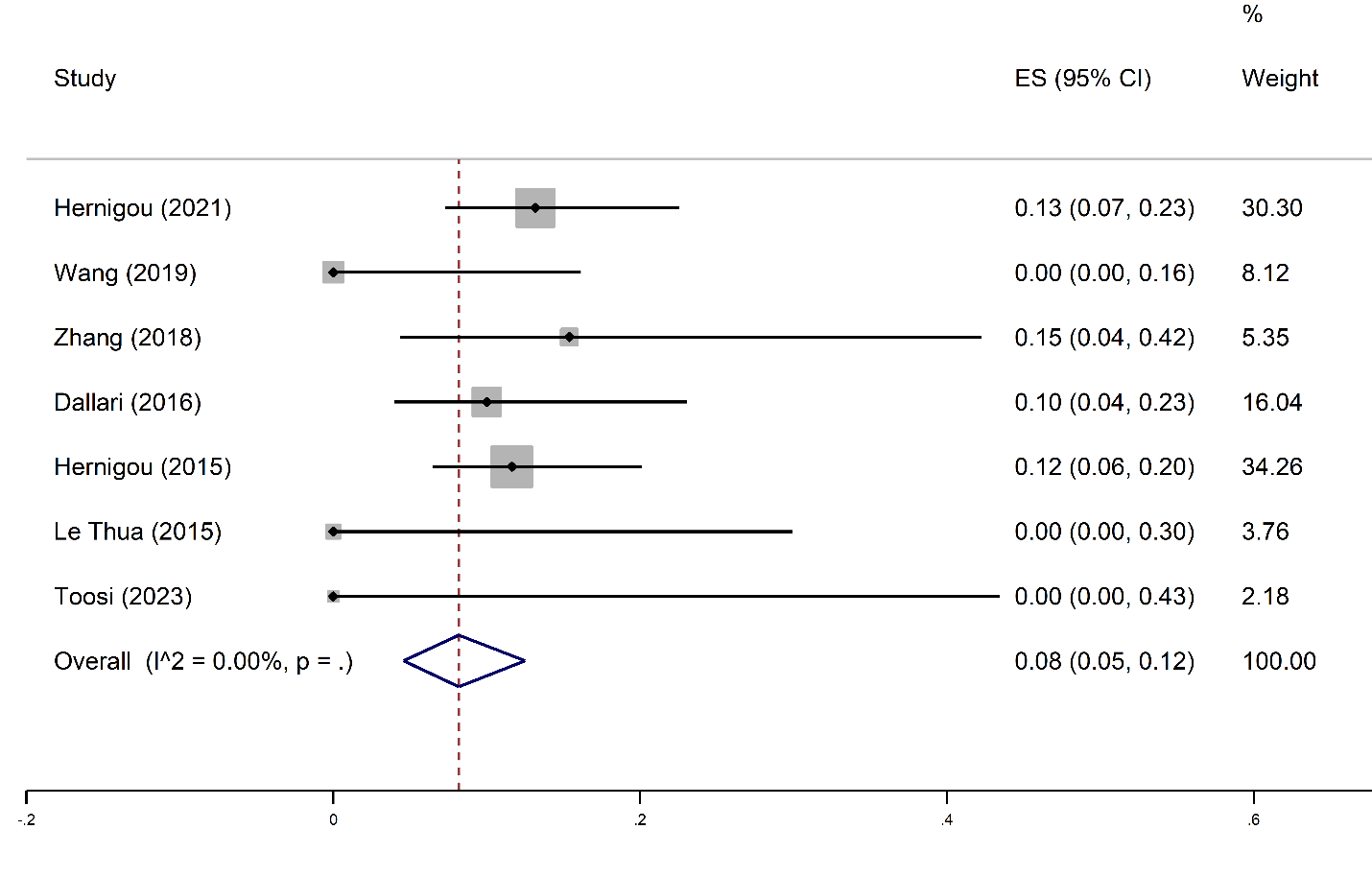


Fig. S2. The rate of complications in the group receiving only autograft (without MSCs)
